# Comparative Omics Analysis of Four Grape Varieties and Exploration of Their Anthocyanin Synthesis Mechanisms

**DOI:** 10.3390/genes16080955

**Published:** 2025-08-13

**Authors:** Kai Zhang, Liyang Zhao, Yanfeng Li

**Affiliations:** Institute of Vegetables, Tibet Academy of Agricultural and Animal Husbandry Sciences, Lhasa 850032, China; zhangkai79@126.com (K.Z.); y13618943375@163.com (L.Z.)

**Keywords:** *Vitis vinifera* L., transcriptomics, metabolomics, anthocyanin biosynthesis, gene expression

## Abstract

**Background**: *Vitis vinifera* L. exhibits diverse varietal traits influencing fruit quality and stress tolerance. The summer black grape (Xiahei), known for its superior tolerance to abiotic stress and intense pigmentation, was hypothesized to possess distinct metabolic and genetic profiles, particularly in flavonoid and anthocyanin biosynthesis. This study aimed to elucidate the metabolic and molecular basis underlying these phenotypic traits by comparing carbohydrate composition and metabolomic and transcriptomic profiles of four grape varieties (summer black, flame seedless, black grape, and red milk). **Methods:** Grapes were consistently sampled five days after full maturity, and metabolites were analyzed using UPLC-MS/MS and GC-MS, while transcriptome analysis employed RNA sequencing followed by qRT-PCR validation. **Results:** The results demonstrated that carbohydrate content was similar among all grape varieties, whereas the summer black grape showed significantly higher levels of flavonoids, particularly anthocyanins such as delphinidin-3-*O*-glucoside, cyanidin-3-*O*-glucoside, and pelargonidin-3-*O*-glucoside. Metabolomic analyses revealed substantial enrichment of metabolites involved in flavonoid biosynthesis pathways, in agreement with transcriptomic data showing significant upregulation of key regulatory genes (CHS, DFR, and ANS) specific to anthocyanin biosynthesis. These findings suggest that the pronounced anthocyanin accumulation in summer black grape contributes to its distinctive dark pigmentation and enhanced resistance to abiotic stresses compared to other varieties. **Conclusion:** This study provides novel insights into the molecular and metabolic mechanisms driving anthocyanin accumulation in summer black grapes, which could inform future breeding programs aimed at improving grape resilience.

## 1. Introduction

Grape (*Vitis vinifera* L.), a tall, entwined vine belonging to the grape family and genus *Vitis*, is a delicious and nutritious fruit with a long history of cultivation [1]. Originating in the West Asian region, grapes were introduced to China during the Western Han Dynasty and are now cultivated widely across multiple provinces, including Hebei, Henan, and Shanxi. Globally, grapes remain one of the most produced fruits [2]. The primary sugars in grapes, including glucose, fructose, and sucrose, influence the sweetness and taste of wine. Grapes are known for their effects in nourishing Qi and blood, relaxing tendons and collaterals, and promoting urination [3]. Regarded as precious fruits and among the top four fruits globally, grapes can be consumed fresh or used to make wine, with their fruits, roots, and leaves also serving medicinal purposes. In years with sufficient light and moderate temperatures, grapes can accumulate higher levels of sugar and flavor compounds, leading to richer, fuller, and higher-quality wines [3,4]. In summary, grapes are not only delicious and nutritious but also versatile, with a wide range of uses.

Xiahei grape (summer black grape) is notable for its strong disease resistance, early maturity, high yield, and excellent taste. Young branches appear yellow-green, while mature shoots turn reddish-brown. Large, light green leaves are dense and hairy, featuring shallow, jagged edges. With hormone treatment, grape clusters assume cylindrical or conical shapes with closely packed berries weighing around 8 g each. The fruit has thick, dark blue to black skin with a thick bloom, firm and crisp flesh with light red juice, and a sweet taste. Notably, the summer black grape (also referred to here and in some texts as Xiahei) demonstrates more than 85% bud differentiation and germination, high sugar content, and excellent storage and transport properties, including a reduced tendency to crack under pressure [5]. Hongru (Red Earth) is a red grape variety developed through hybridization at California State University and introduced to China in 1987. It has shown exceptional performance in North and Northwest China and is characterized by its high fruit quality, late maturity, and robust storage and transportation capabilities. This variety features large, elongated, conical clusters with an average weight of 850 g and under optimal conditions may reach up to 2500 g. The berries are round or oval, with moderate elasticity and even distribution. The thick skin and dark red color complement the hard, crisp flesh, which can be sliced thinly. The grapes have a sweet, pure flavor with soluble solids exceeding 16.5%, and they maintain high quality without juice leakage when cut. The long fruit stalks are securely attached, and the thick fruit brush exhibits strong tensile strength, reducing the risk of berry loss. This variety is well-suited for long-distance transport and extended storage, remaining viable until March of the following year. Flame seedless, also known as Frey seedless, red light seedless, or red pearl, originated from the United States. It was selected through hybridization at the at the Fresno Horticultural Experiment Station (USA) and introduced to China in 1983. The mature branches are dark reddish-brown, with evenly spaced internodes and bisexual flowers. This variety produces large, cone-shaped clusters weighing between 680 and 890 g, with some reaching up to 1500 g. The berries are moderately dense, nearly round, and have a vivid red to purplish-red hue, with an average weight of 3 to 3.5 g each. red milk, a mid- to late-maturing Eurasian variety, is renowned for its distinctive appearance and exceptional quality. The variety features dense clusters with slender berries and pointed fruit tips. Individual berries weigh between 9 and 11 g, and clusters typically range from 500 to 700 g. The flesh is hard, crisp, and extremely sweet, with a fragrant, refreshing flavor. Handling this variety leaves a sticky residue on the fingers, indicating high sugar content.

Anthocyanins, essential flavonoid-derived pigments in grapes, contribute significantly to fruit coloration and stress tolerance. Despite considerable research on grape anthocyanin biosynthesis [6,7], varietal differences, particularly regarding stress resilience mechanisms, remain poorly understood [8]. In addition, anthocyanins exhibit various beneficial biological activities in humans, such as antioxidative, anticancer, anti-inflammatory, and anti-aging effects [9]. They can reduce low-density lipoprotein accumulation and prevent hypertension, hyperlipidemia, and hyperglycemia by regulating vascular endothelial cells and controlling the signal transduction pathways of smooth muscle contraction [10,11,12]. Anthocyanins also inhibit fat formation and assist in the decomposition of excess fat, thus contributing to a blood-fat-reducing effect. Enhancing the ornamental value of broad bean flowers as well as developing petal essential oils and petal beverages are potential ways to increase their economic value [13]. This study hypothesizes that the summer black grape exhibits distinct metabolic and genetic adaptations that promote anthocyanin accumulation and stress tolerance, distinguishing it from other varieties. Exploring these adaptations could offer new insights into grape breeding programs aimed at enhancing resilience to abiotic stresses.

In the present study, carbohydrate content was measured, and the regulatory mechanisms underlying anthocyanin biosynthesis were investigated using transcriptomic and metabolomic approaches. Focusing on the Xiahei grape, differently expressed metabolites and genes were identified that distinguish it from other varieties, with notable enrichment in the flavonoid and anthocyanin biosynthesis pathways. We further constructed a grape anthocyanin regulatory pathway and observed significant upregulation of both metabolites and regulatory genes in summer black grapes. These findings may explain their darker coloration and superior stress tolerance. Taken together, the results presented here provide a valuable reference for understanding anthocyanin biosynthesis in grapes and will facilitate the breeding of high-yielding grape cultivars with robust resistance to abiotic stress [14].

## 2. Materials and Methods

### 2.1. Sampling and Preparing

**Figure 1:**
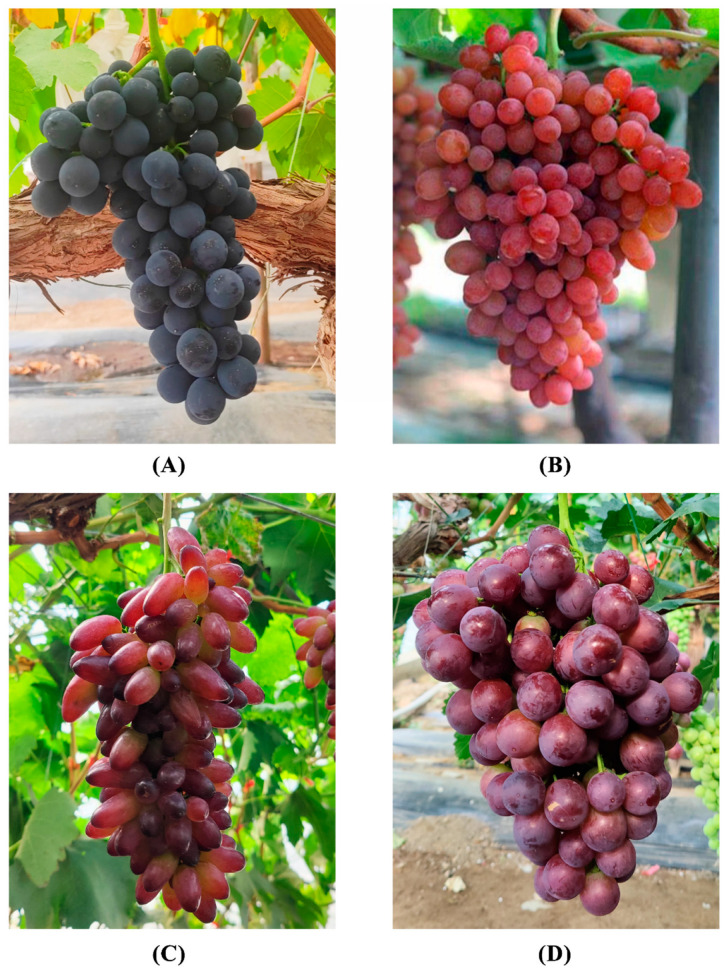
Characteristics of *V. vinifera* samples with different varieties: (**A**) summer black grape, (**B**) flame seedless grape, (**C**) black grape, (**D**) red milk grape.

The grapevines used for this study were cultivated under standard field conditions in a high-efficiency solar greenhouse located in the Lhasa National Agricultural Science and Technology Park (latitude 29°40′ N, longitude 116°29′ E, elevation 3648.7 m). The region is characterized by a temperate plateau semi-arid monsoon climate with cool temperatures, large diurnal temperature variations, and small annual temperature fluctuations. The annual mean temperature ranges from 6 °C to 7.5 °C, with average daily maximum and minimum temperatures of 15.4 °C and 0.7 °C, respectively. The annual accumulated temperature above 0 °C is 2887 °C. Annual precipitation averages 450 mm, over 90% of which occurs between June and August, while spring and early summer droughts are common. The region has abundant solar radiation, receiving an annual total of 3021 h of sunshine and 7745 MJ/m^2^ of total radiation. The effective radiation above 0 °C is 2700 MJ/m^2^ annually. The frost-free period spans approximately 135–145 days (Figure 1).

Grapevines were drip-irrigated using a water-fertilizer integrated system. The soil type was sandy loam, which exhibits good tillage properties and is rich in microelements. The soil contained approximately 124.5 mg/kg available nitrogen, 0.182% total phosphorus, 2.35% total potassium, and 1.79% organic matter. Planting density was maintained at 1 m × 2 m, and vines were originally planted in 2014.

Four grape varieties—summer black, flame seedless, black grape, and red milk grape—were selected at five days post-maturity. Temperature during phenological stages was carefully controlled: 16–22 °C during budburst, 22–26 °C during flowering, 24–26 °C during fruit expansion, and 26–29 °C during ripening. For each variety, five plants were selected, and samples were collected in triplicate as independent biological replicates. Fresh berry tissues were immediately frozen in liquid nitrogen and stored at −80 °C until use.

Before RNA extraction, all Eppendorf tubes and pipette tips were pre-treated with 0.1% DEPC water for 24 h then autoclaved and dried. Total RNA was extracted using TRIzol reagent according to the manufacturer’s instructions. RNA quality and integrity were assessed using an Agilent 2100 Bioanalyzer (Agilent Technologies, Santa Clara, CA, USA), and only samples with RNA Integrity Number (RIN) values ≥ 7.0 were used for downstream analysis.

For metabolomic profiling, internal standards (e.g., ribitol) were used for normalization of sugar content. Standard curves were prepared for each sugar using known concentrations, and calibration curves achieved R^2^ values ≥ 0.995. The abundance of each metabolite was quantified using external calibration and normalized to the internal standard. Values reported represent biological replicates unless otherwise stated.

### 2.2. Metabolite Identification and Quantification

#### 2.2.1. UPLC-MS/MS and GC-MS Conditions

Metabolite characterization was conducted using a Nexera X2 UPLC system (Shimadzu, Kyoto, Japan) coupled with a 4500 Q TRAP mass spectrometer (Agilent Technologies, Santa Clara, CA, USA). Chromatographic separation was achieved on an Agilent SB-C18 column (Agilent Technologies, Santa Clara, CA, USA) (1.8 µm, 2.1 mm × 100 mm) maintained at 40 °C. The mobile phases consisted of solvent A (0.1% formic acid in water) and solvent B (0.1% formic acid in acetonitrile). The flow rate was set to 0.35 mL/min, and the injection volume was 4 µL. The elution gradient is detailed below in Table 1.

Mass spectrometric data were acquired in both positive and negative ion modes using Analyst 1.6.3 software (AB Sciex, Framingham, MA, USA). The system was operated in multiple reaction monitoring (MRM) mode using nitrogen as the collision gas, with instrument parameters optimized accordingly for QQQ scans.

For sugar measurement, an 8890 GC (Agilent) equipped with a 5977B detector (Agilent Technologies, Santa Clara, CA, USA) was utilized. Samples were introduced at a 5:1 split ratio by injecting 1 µL each onto a DB-5MS column (30 m × 0.25 mm i.d. × 0.25 µm film thickness). Helium served as the carrier gas at 1 mL/min. The oven was programmed to hold 160 °C for 1 min, increase to 200 °C at 6 °C/min, rise to 270 °C at 10 °C/min, then to 300 °C at 5 °C/min, and finally reach 320 °C at 20 °C/min for 5.5 min. Ion source temperature was kept at 230 °C, with the transfer line at 280 °C, and data were acquired in selective ion monitoring mode.

#### 2.2.2. Qualitative and Quantitative Analyses of Metabolites

Data were processed with Analyst v1.6.3 by applying total ion current (TIC) monitoring for quality control (QC) samples and using MRM-based multi-substance extracted ion current (XIC) to detect metabolites, as illustrated in Figure S1. Each peak was identified based on characteristic ions and their signal intensities in comparison with a local metabolism database (Metware, Wuhan, China). Chromatographic peaks were integrated and corrected with MultiaQuant, and peak area was taken as an indicator of metabolite abundance.

Standard solutions (0.001–50 μg/mL) were prepared, and corresponding peak intensities were measured. Calibration curves were generated by plotting the ratio of the external-standard concentration to the internal-standard concentration against the ratio of the external-standard peak area to the internal-standard peak area. Linear equations and determination coefficients (R^2^) are summarized in Table 2.

#### 2.2.3. Significantly Regulated Metabolites

Metabolites were considered significantly regulated if they satisfied the thresholds VIP ≥ 1 and |log2FC| ≥ 1. VIP values were derived from OPLS-DA models built using MetaboAnalystR, which also provided score plots and permutation tests (200 permutations) to guard against overfitting [15]. Prior to OPLS-DA, the dataset was log2-transformed and mean-centered. Principal component analysis (PCA) was then performed via the prcomp function in R (Version 4.1.1, www.r-project.org (accessed on 16 September 2024)) following unit variance scaling.

### 2.3. RNA-Seq and Data Analysis

#### 2.3.1. cDNA Library Construction and RNA Sequencing

The total RNA was isolated from grape berry skin (pericarp) tissue, followed by reverse transcription to obtain double-stranded cDNA. The purified cDNA underwent end repair, A-tailing, and adapter ligation, and ~200 bp fragments were isolated with AMPure XP beads. The final cDNA libraries were sequenced at Wuhan Beina Technology Co., Ltd. (Wuhan, China) on an Illumina platform. Quality control of raw reads was performed with Fastp [16] (https://github.com/OpenGene/fastp (accessed on 24 September 2024)), which discarded low-quality sequences and removed adapters, yielding clean reads for further analysis.

#### 2.3.2. Transcript Assembly, Functional Annotation, and Differential Gene Expression

Transcript assembly was conducted via Trinity v2.6.6 with the parameter “--min_kmer_cov 2” [17]. Of each gene’s transcripts, the longest was designated as the unigene, resulting in 25,736 unigenes. Annotations were assigned by comparing these unigenes against the NCBI nonredundant (NR), UniProt, KEGG, and GO databases [18,19].

Differentially expressed genes were identified using DESeq2 (Version 3.18.x), selecting those with a false discovery rate (FDR) < 0.05 and |log2FC| > 1 as significant. Structural and regulatory genes associated with anthocyanin biosynthesis were emphasized among the significant results. Each candidate gene was compared to hidden Markov model (HMM) domains from the Pfam database [20] to discern structural features and infer potential roles in anthocyanin formation.

### 2.4. Verification of RNA Sequences via RT-qPCR

Verification of differentially expressed genes linked to anthocyanin biosynthesis was carried out using RT-qPCR. Total RNA was extracted with the RNAprep Pure Plant Kit (Tiangen, Beijing, China), followed by reverse transcription using HiScript IV RT SuperMix for qPCR (+gDNA wiper) (Novozymes, Nanjing, China). The resulting first-strand cDNA was diluted fivefold and used as the template for qPCR reactions on a BIO-RAD CFX96 system, employing ChamQ Universal SYBR qPCR Master Mix (Novozymes). Gene-specific primers were designed through IDT (https://sg.idtdna.com/PrimerQuest (accessed on 28 September 2024)). VvActin and VvGAPDH served as internal reference genes [21]. For qRT-PCR validation, all primer sequences are provided in Supplementary Table S1. The thermal cycling program was set as follows: initial denaturation at 95 °C for 30 s, followed by 40 cycles of 95 °C for 10 s and 60 °C for 30 s. Gene expression levels were normalized using appropriate housekeeping genes, and results were presented as mean values from three biological replicates.

## 3. Results

### 3.1. Multivariate Analysis of the Metabolome

#### 3.1.1. Widely Targeted Metabolome Profiles

**Figure 2:**
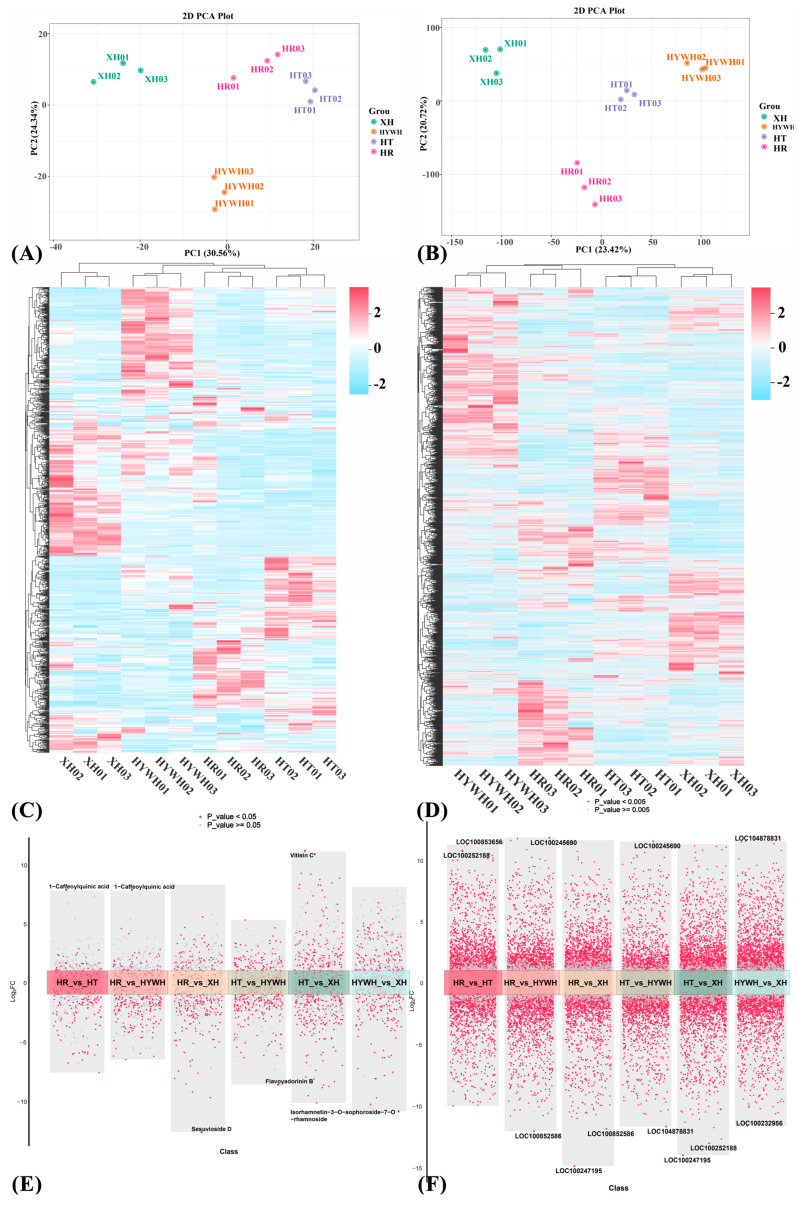
Metabolomic and transcriptomic analysis of *V. vinifera* samples with different varieties. XH represents Xiahei grape; HYWH represents Huoyanwuhe grape; HT represents Heiti grape; HR represents Hongru grape. Principal component analysis of metabolome (**A**) and transcriptome (**B**). Cluster heatmap of *V. vinifera* metabolome (**C**) and transcriptome (**D**) with different varieties. Multiple volcano plots of differential metabolites (**E**) and genes (**F**). The part on the horizontal axis of the coordinate axis represents the upregulated metabolites or genes, while the part below the horizontal axis represents the downregulated metabolites or genes.

UPLC-MS analyses revealed that different grape flower groups contain abundant flavones in Table S2. To examine global metabolic differences, principal component analysis (PCA) and hierarchical clustering of both metabolites and genes were performed [22]. These results revealed that samples within each group exhibited similar metabolic and transcriptomic profiles, clustering separately in Figure 2A–D. Metabolites and differentially expressed genes (DEGs) with a fold change >2 or <0.5—meaning they had either more than doubled or declined to less than half—were considered significantly altered between control and experimental groups. A volcano plot was generated to visualize these relative differences in metabolite content and their statistical significance, as shown in Figure 2E,F.

#### 3.1.2. Qualitative and Quantitative Analysis of Carbohydrates

**Figure 3:**
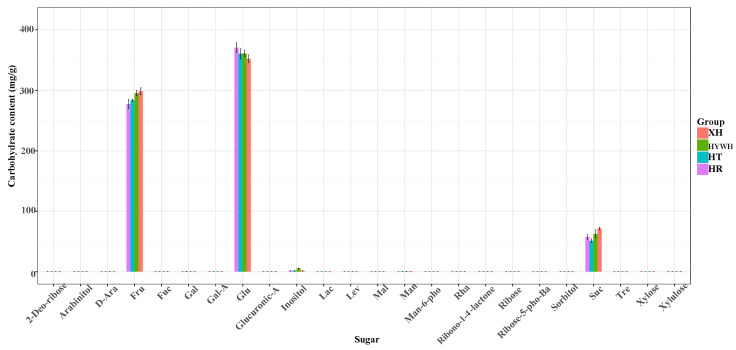
The quantitative histogram plot of carbohydrate contents. Summer black grape (XH), flame seedless grape (HYWH), black grape (HT), and red milk grape (HR). A one-way analysis of variance (ANOVA) was performed on the statistical data, revealing significant differences in the contents of various sugars among different groups. Notably, D-fructose (Fru) exhibited a high F-value of 49.3224 with a *p*-value of 0.0000, indicating a statistically significant difference. Similarly, sucrose (Suc) showed an F-value of 6.3374 and a *p*-value of 0.0000. D-galactose (Glu) also demonstrated a significant variation, with an F-value of 52.7433 and a *p*-value of 0.0000.

Carbohydrates, comprising carbon, hydrogen, and oxygen, can be hydrolyzed into polyhydroxyaldehydes or ketones. They make up more than 50% of Earth’s biomass by dry weight, and 85–90% of plant dry weight can be attributed to sugar polymers. As essential organic molecules, carbohydrates are involved in numerous physiological and developmental processes, such as cell signaling, immune defense, metabolic regulation, and fertilization. Using a GC-MS platform, a targeted sugar analysis method was developed to detect and quantify the 24 distinct sugar substances in Figure 3. Among these, glucose (352.29–370.53 mg/g), D-fructose (277.08–298.48 mg/g), and sucrose (57.42–71.30 mg/g) were the primary contributors to sweetness. All four grape varieties exhibited high sweetness levels with only slight differences in overall sugar content.

### 3.2. Metabolome-Based Comparison of the Four Grape Varieties

**Figure 4:**
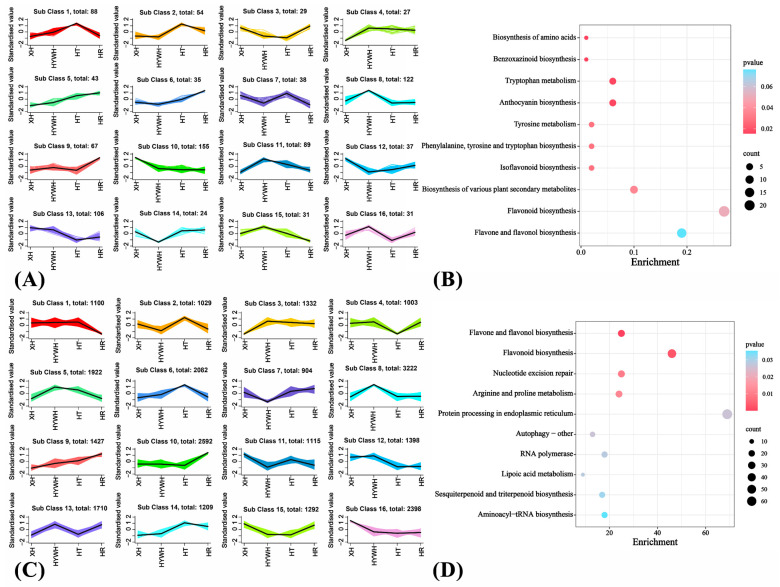
(**A**) The Kmeans analysis of metabolites. (**B**) The KEGG enrichment results of differential metabolites. (**C**) The Kmeans analysis of genes. (**D**) The KEGG enrichment results of differential genes.

Xiahei (summer black) grapes are notable for their disease resistance, high yield, early maturity, storage tolerance, and desirable flavor. Nevertheless, the genetic or metabolic basis of their strong stress resistance is not fully understood. Therefore, Xiahei served as the main subject in subsequent analyses. Using datasets from all four varieties, differential metabolites and genes were identified based on standardized screening criteria. After unit variance scaling (UV), K-means clustering divided these metabolites into 16 groups based on the differences in their relative contents among different grape varieties. In Xiahei, Groups 10 and 12 contained metabolites that were upregulated relative to other varieties, whereas Group 4 included metabolites that were downregulated. KEGG enrichment of these clusters pointed to pathways linked to defense and stress tolerance, such as amino acid synthesis, benzoxazinoid biosynthesis, and anthocyanin synthesis (Figure 4, Table S4).

Genes were similarly grouped into 16 clusters according to their expression trends. Group 16 comprised genes that were highly expressed in Xiahei, whereas Group 3 included genes downregulated in Xiahei. KEGG analysis of these gene clusters highlighted enrichment in flavonoid and flavonol biosynthesis such as myricetin, dihydromyricetin, and epigallocatechin as well as upstream segments of the anthocyanin synthesis pathway including delphinidin-3-*O*-(6″-*O*-p-coumaroyl)glucoside. Taken together, these metabolite and gene enrichment results suggest that Xiahei accumulates more flavonoids and related derivatives—particularly anthocyanins—than the other grape varieties, potentially explaining its robust stress resistance.

### 3.3. Beery Coloring and Anthocyanin Accumulation in the Grape Varieties

**Figure 5:**
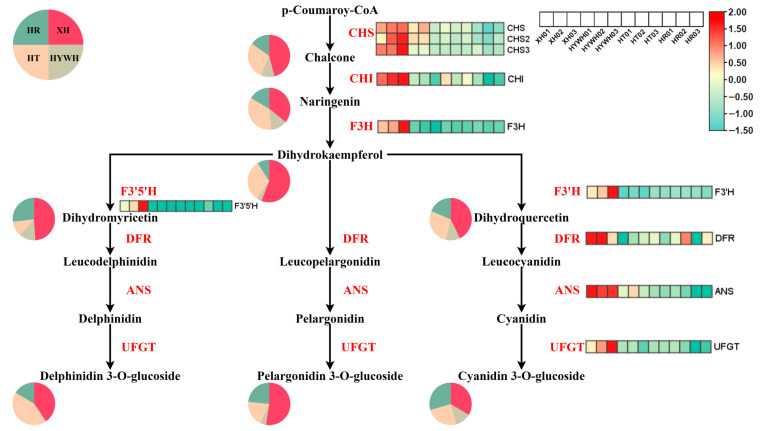
The expression levels of structural genes and anthocyanin content involved in the anthocyanin biosynthesis pathway were examined in different colored flowers of *V. vinifera*. The heatmap was utilized to illustrate the expression patterns of these structural genes in four *V. vinifera* cultivars, and the pie chart represents the anthocyanin content, with colors ranging from blue-green to red, indicating low to high expression levels of the respective genes. The enzymes represented in this pathway are as follows: chalcone synthase (*CHS*), chalcone isomerase (*CHI*), flavanone 3-hydroxylase (*F3H*), flavonoid 3′-hydroxylase (*F3′H*), flavonoid 3′,5′-hydroxylase (*F3′5′H*), dihydroflavonol 4-reductase (*DFR*), anthocyanidin synthase (*ANS*), UDP-glucose:flavonoid 3-*O*-glucosyltransferase (*UFGT*), and glutathione S-transferase (GST).

Following a thorough examination of the documented anthocyanin biosynthesis pathway and its associated gene identifications, a pathway illustration incorporating the expression heatmap for every structural gene pertinent to anthocyanin biosynthesis of *V. vinifera* was devised (Figure 5). Fundamental to the early enzymatic reactions regulating anthocyanin biosynthesis are the structural genes *CHS* and *CHI* [23]. Additionally, *F3H* catalyzes the conversion of naringenin to dihydrokaempferol, a crucial precursor for multiple anthocyanin branches [24]. The expression patterns of *F3′H* and *F3′5′H* further determine the composition of specific anthocyanin molecules [25,26]. Meanwhile, *DFR* and *ANS* exhibit varied catalytic capacities for different substrates in the pathway [27], and a highly expressed *DFR* gene was identified in *V. vinifera*. In later stages, *UFGT* enzymes convert volatile anthocyanins into stable forms, thus finalizing anthocyanin biosynthesis [28]. Comparisons among the four grape varieties showed that the genes and metabolites linked to anthocyanin production were consistently upregulated in Xiahei. These heightened flavonoid and anthocyanin levels may contribute to Xiahei’s notable stress resistance.

**Figure 6:**
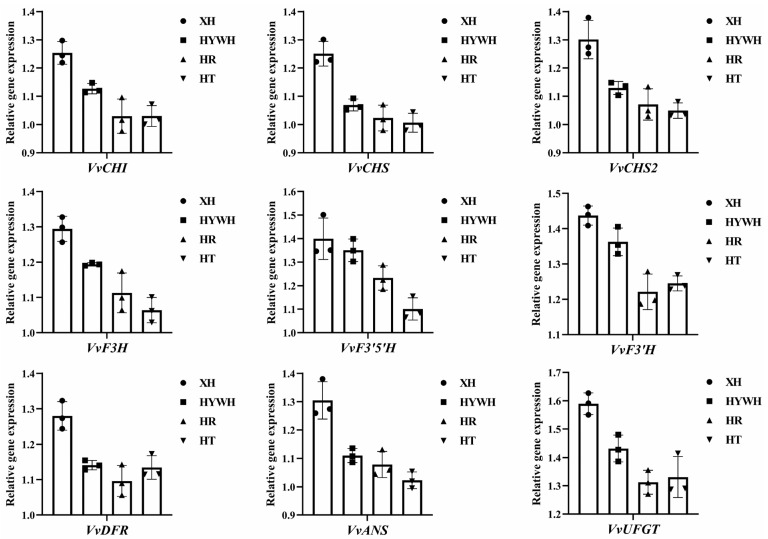
The relative gene expression in the anthocyanin pathway. A one-way analysis of variance (ANOVA) was performed on the statistical data, revealing significant differences in the expression of regulated genes among different groups.

To validate the transcriptomic findings, we conducted qRT-PCR on anthocyanin pathway genes in Xiahei grapes. As illustrated in (Figure 6), key genes for anthocyanin production were indeed overexpressed, confirming the results obtained from our transcriptomic analysis.

## 4. Discussion

This study confirms that all four grape varieties analyzed—summer black, flame seedless, black, and red milk—possess high sugar content, with glucose (352.29–370.53 mg/g), d-fructose (277.08–298.48 mg/g), and sucrose (57.42–71.30 mg/g) as the dominant sugars. These results are consistent with previous reports on *Vitis vinifera* cultivars, where glucose and fructose have been identified as the major soluble sugars contributing to grape sweetness and consumer acceptability [29]. Notably, summer black grapes exhibited significantly higher concentrations of flavonoid metabolites, especially anthocyanins, compared to the other varieties. The elevated anthocyanin levels not only explain the darker pigmentation of the fruit but are also strongly associated with improved stress resistance, which can extend shelf life and facilitate transport. Similar patterns have been observed in other dark-colored grape varieties, such as Cabernet Sauvignon and Concord, which also show high anthocyanin levels and enhanced resistance to environmental stressors like UV radiation and temperature extremes [30]

Anthocyanins, synthesized through the flavonoid biosynthesis pathway, are essential pigments responsible for the coloration of plant flowers and fruits while also serving as protective compounds against environmental stressors, such as ultraviolet radiation, drought, and oxidative damage [31]. In this study, targeted metabolomics identified four primary anthocyanins in grapes, with delphinidin-3-*O*-glucoside, cyanidin-3-*O*-glucoside, and pelargonidin-3-*O*-glucoside being the most influential. Pelargonidin-3-*O*-glucoside typically imparts an orange hue, cyanidin-3-*O*-glucoside contributes a red coloration, and delphinidin-3-*O*-glucoside appears blue or purple. Accordingly, the deep purple seed coat of summer black grapes reflects the high accumulation of anthocyanins, which in turn supports their enhanced stress resilience [32]. This aligns with reports from other plant species such as *Vaccinium* spp. (blueberries) and *Fragaria* spp. (strawberries), where these compounds contribute to coloration and offer antioxidant protection [33].

Previous research has established that anthocyanin biosynthesis depends on a set of early biosynthesis genes (e.g., *CHS*, *CHI*, and *F3H*) and anthocyanin-specific genes (e.g., *F3′H*, *F3′5′H*, *DFR*, *ANS*, and *UFGT*) [34]. Numerous transcription factors, particularly those belonging to the *MYB* family such as *MYB4A* and *MYB24*, also regulate this complex pathway [35]. In this study, *CHS*, *DFR*, and *ANS* were significantly upregulated in summer black grapes compared with the other varieties. Strong *CHS* expression drives the production of intermediates like chalcone and naringin, which are pivotal for anthocyanin biosynthesis. Dihydrokaempferol functions as a pivotal precursor and branching node in the pathway across various plant species, while *DFR* catalyzes multiple substrate types, ultimately producing leucodelphinidin, leucopelargonidin, and leucocyanidin. These intermediates are subsequently converted by *ANS* into delphinidin, pelargonidin, and cyanidin, respectively. Both *DFR* and *ANS* showed markedly high expression in summer black grapes. In contrast, *F3′5′H* expression was lower than *F3′H*, leading to a shift toward more cyanidin-derived pigments, giving a purplish-red hue in mature berries. Altogether, these gene expression patterns corroborate the high anthocyanin content observed in summer black grapes and help explain their distinctive coloration and robust stress resistance [36].

Furthermore, the anthocyanin-related stress tolerance of summer black grapes mirrors findings in other high-anthocyanin cultivars, such as “Yan73” and ”Muscat Hamburg”, where higher anthocyanin concentrations have been associated with enhanced drought and UV resistance [37]. These comparative data suggest that the metabolic reprogramming observed in summer black grapes is not unique but reflects a conserved adaptive strategy among pigmented fruits.

The role of MYB transcription factors, which are central regulators of anthocyanin biosynthesis, is also well-documented across various plant taxa. While our study primarily focused on structural gene expression, future work examining the expression of MYB, bHLH, and WD40 genes in summer black grapes could further clarify the upstream regulatory mechanisms, as demonstrated in apples and roses [38].

From a breeding perspective, the superior performance of summer black grapes in terms of pigmentation and stress tolerance makes them a promising genetic reservoir. Their gene expression and metabolite profile offer valuable markers for introgression into new varieties. Moreover, the anthocyanin accumulation observed may serve as a visual and molecular indicator for selecting stress-resilient cultivars, echoing strategies used in rice and sorghum breeding programs [39].

The integration of metabolomics and gene expression data in this study not only reaffirms the metabolic richness of summer black grapes but also contributes to a broader understanding of flavonoid-mediated stress resilience in grapes and other fruit crops. Subsequently, based on the research foundation of this article, further studies on abiotic stress in grapes can be carried out, including reactive oxygen species (ROS)-clearing enzymes, osmotic fluid accumulation, hormone signal transduction, and stress response transcription factors. Continued comparative studies across grape cultivars and related species will further elucidate the evolutionary and functional significance of anthocyanin diversity in horticultural adaptation.

## 5. Conclusions

In conclusion, this study provides significant insight into the metabolic and genetic features that distinguish summer black grapes from other varieties. By confirming the higher accumulation of anthocyanins and the upregulation of key biosynthetic genes, this research offers valuable perspectives on the molecular basis of stress resistance in grapes. These findings not only enrich our understanding of anthocyanin biosynthesis but also lay the groundwork for breeding programs aimed at developing stress-resistant, high-quality grape cultivars.

## Figures and Tables

**Table 1:** Sample determination process.

Time (min)	Solvent A (%)	Solvent B (%)	Description
0	95	5	Initial condition
0.00–9.00	From 95% to 5%	From 5% to 95%	Linear gradient change
9.00–10.00	5	95	Maintained at high organic phase
10.00–11.10	From 5% to 95%	From 95% to 5%	Linear return to initial composition
11.10–14.00	95	5	Column re-equilibration

**Table 2:** Linear equation table of standard sugar substances.

Compound	Class	RT	Equation
2-Deoxy-d-ribose (2-Deo-ribose)	monosaccharide	3.504	y = 0.338298 x + 7.618096 × 10^−5^
d-Xylose (Xylose)	monosaccharide	4.239	y = 0.694375 x + 5.218014 × 10^−5^
d-Arabinose (d-Ara)	monosaccharide	4.311	y = 0.901696 x + 1.438163 × 10^−4^
d-Ribose (Ribose)	monosaccharide	4.439	y = 0.864945 x + 5.076758 × 10^−4^
d-Xylulose (Xylulose)	monosaccharide	4.44	y = 1.107687 x + 0.003011
d-Ribono-1,4-lactone (Ribono-1-4-lactone)	monosaccharide	4.571	y = 0.286528 x + 5.477765 × 10^−5^
Xylitol	monosaccharide	4.761	y = 1.311257 x + 4.677106 × 10^−4^
l-Levoglucosan (Lev)	monosaccharide	4.871	y = 0.222450 x − 3.409670 × 10^−5^
l-Rhamnose (Rha)	monosaccharide	4.921	y = 0.590455 x + 2.054933 × 10^−4^
Arabinitol	monosaccharide	4.922	y = 1.200010 x + 6.877416 × 10^−4^

## Data Availability

The datasets used and/or analyzed during the current study are available from the corresponding author on reasonable request.

## Supplementary Materials

The following supporting information can be downloaded at https://www.mdpi.com/article/10.3390/genes16080955/s1: Figure S1: Identification of secondary metabolites of *V. vinifera* (A shows detection of TIC overlay by QC sample negative mass spectrometry, and B shows detection of TIC overlay by QC sample positive mass spectrometry); Table S1: Supplementary file of secondary metabolome data; Table S2: Supplementary file of secondary metabolome data; Table S3: Kmeans results of secondary metabolome data. Table S4: Kmeans results of transcriptome data.

## Abbreviation

LC-MS/MS	Liquid chromatography–tandem mass spectrometry
