# Supplementary material for: Comparative Omics Analysis of Four Grape Varieties and Exploration of Their Anthocyanin Synthesis Mechanisms
Source: Genes (Basel). 2025 Aug 13;16(8):955. doi: 10.3390/genes16080955 (PMC12386037; doi:10.3390/genes16080955)
Supplement: Supplementary file 1 [file genes-16-00955-s001.zip › genes-3779355-supplementary/20240825-Supplemental fiure.pdf]

Figure S1. Identification of secondary metabolites of *V. vinifera* (The A shows detection of TIC overlay by QC sample negative mass spectrometry, and B shows detection of TIC overlay by QC sample postive mass spectrometry.).

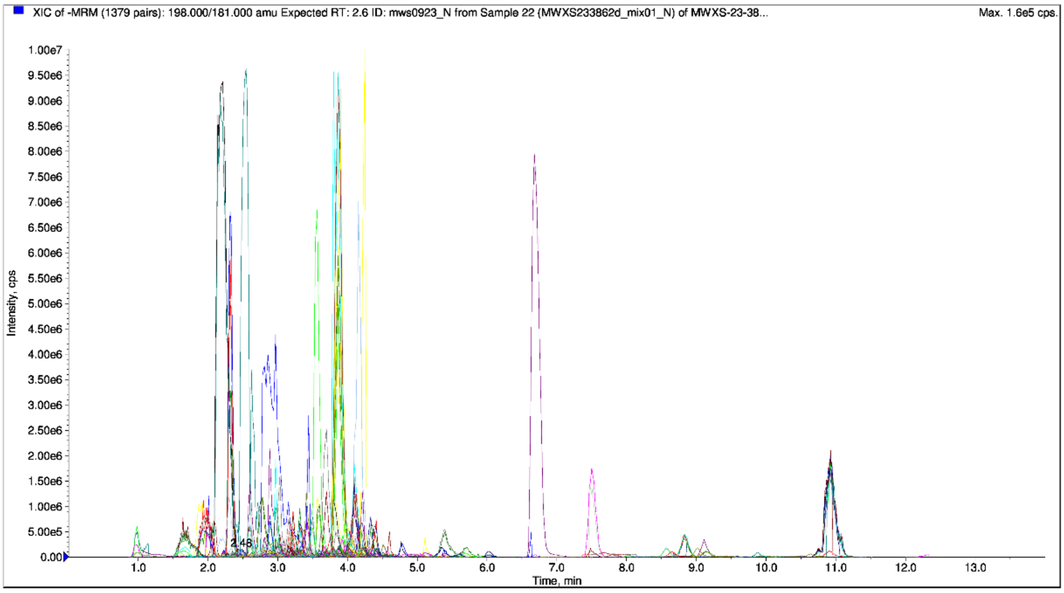

(A)

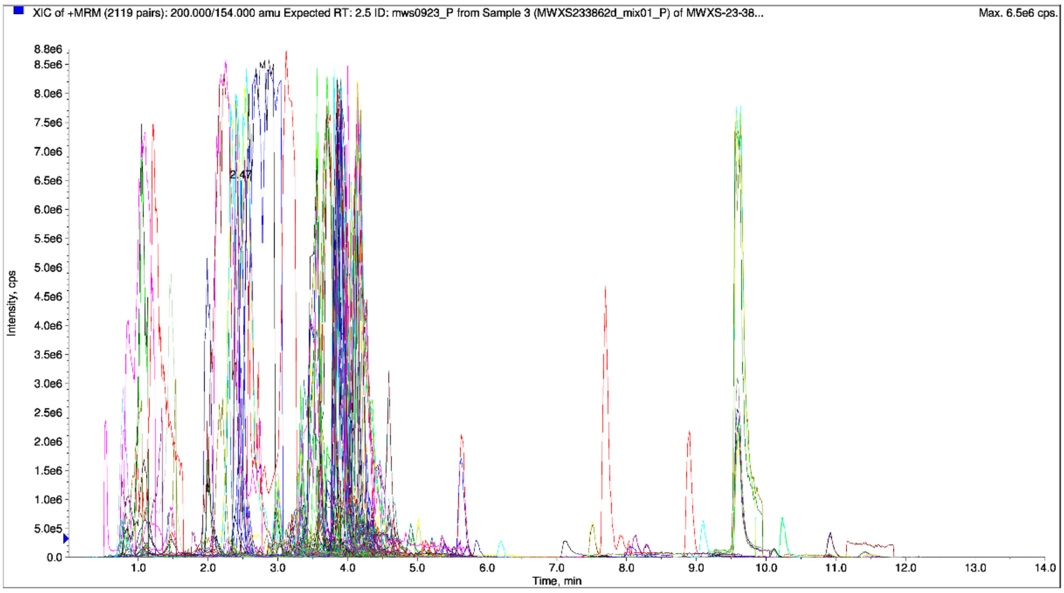

(B)
